# Evaluating ChatGPT-4’s performance as a digital health advisor for otosclerosis surgery

**DOI:** 10.3389/fsurg.2024.1373843

**Published:** 2024-06-05

**Authors:** Samil Sahin, Burak Erkmen, Yaşar Kemal Duymaz, Furkan Bayram, Ahmet Mahmut Tekin, Vedat Topsakal

**Affiliations:** ^1^ Private Practitioner, Istanbul, Türkiye; ^2^ Umraniye Research and Training Hospital, University of Health Sciences, Istanbul, Türkiye; ^3^ Department of Otolaryngology and Head & Neck Surgery, Vrije Universiteit Brussel, Brussels Health Care Center, Brussels, Belgium

**Keywords:** ChatGPT in healthcare, otosclerosis surgery information, AI-based patient education, readability of health information, DISCERN scoring in AI responses

## Abstract

**Purpose:**

This study aims to evaluate the effectiveness of ChatGPT-4, an artificial intelligence (AI) chatbot, in providing accurate and comprehensible information to patients regarding otosclerosis surgery.

**Methods:**

On October 20, 2023, 15 hypothetical questions were posed to ChatGPT-4 to simulate physician-patient interactions about otosclerosis surgery. Responses were evaluated by three independent ENT specialists using the DISCERN scoring system. The readability was evaluated using multiple indices: Flesch Reading Ease (FRE), Flesch-Kincaid Grade Level (FKGL), Gunning Fog Index (Gunning FOG), Simple Measure of Gobbledygook (SMOG), Coleman-Liau Index (CLI), and Automated Readability Index (ARI).

**Results:**

The responses from ChatGPT-4 received DISCERN scores ranging from poor to excellent, with an overall score of 50.7 ± 8.2. The readability analysis indicated that the texts were above the 6th-grade level, suggesting they may not be easily comprehensible to the average reader. There was a significant positive correlation between the referees’ scores. Despite providing correct information in over 90% of the cases, the study highlights concerns regarding the potential for incomplete or misleading answers and the high readability level of the responses.

**Conclusion:**

While ChatGPT-4 shows potential in delivering health information accurately, its utility is limited by the level of readability of its responses. The study underscores the need for continuous improvement in AI systems to ensure the delivery of information that is both accurate and accessible to patients with varying levels of health literacy. Healthcare professionals should supervise the use of such technologies to enhance patient education and care.

## Introduction

Patients often refer to the internet to obtain medical information (1). Recent technological advances have made the use of artificial intelligence (AI) more available via the internet in many sectors and thus also in the healthcare sector. AI offers significant benefits in decision-making processes in terms of speed and accuracy (2). ChatGPT, an AI chatbot developed by OpenAI, is one of the most effective tools in this field. This tool can have an interactive and fluent exchange of information with users that resembles a dialog, making it a highly suitable tool for quenstion and answer setting such as counseling patients (3).

Otosclerosis, a disease of the otic capsule involving resorption of compact bone and redeposition of spongiotic bone. It most commonly causes conductive hearing loss as a result of stapes fixation (4, 5). Otosclerosis is an autosmomal dominant genetic disease not aquired. The hearing loss is progressive and can be treated with an hearing aid or surgically (6). The purchase of hearing aids are without any risk and restores hearing maximally in contrast to the surgery that contains risks and may restore the hearing loss partially. Although stapedoplasty is a well standerdized procedure with a high succesrate patients should be correctly informed about the risks and outcomes.

Patients can often have many questions and concerns before they can decide for surgical procedures. Having healthcare professionals respond to these concerns with timely and accurate information plays a critical role in improving patient outcomes. However, the contact with healhcare workers alone may not always be enough. AI-based chatbots have the potential to provide patients with the necessary or additional information. In this study, we evaluate how ChatGPT responds to patients' questions prior to otosclerosis surgery. We will evaluate the validity and readability of the robot's answers, to see if there is place for such a tool in healthcare for this type of hearing loss.

## Methods

On October 20, 2023, fifteen hypothetical questions were formulated to simulate a physician-patient consultation on otosclerosis surgery. These questions were developed through a consensus process by a team specialized in otosclerosis, drawing upon their clinical experience. The team selected questions that are frequently encountered in clinical settings, ensuring they accurately reflect the concerns and informational needs of patients considering otosclerosis surgery.

The process of question formulation involved an initial collection of common queries from clinical encounters and medical records, followed by several rounds of discussions. This iterative process allowed for the refinement and validation of the final set of questions, aiming to cover a wide range of topics from surgical risks to post-operative expectations.

Three independent otolaryngology specialists, with no prior knowledge of the question formulation team, were selected to evaluate ChatGPT-4's responses. These experts, chosen for their extensive experience in otosclerosis, assessed the responses independently. Criteria for expert selection included significant clinical experience, scholarly contributions to otosclerosis, and peer recognition. This ensured an objective and comprehensive evaluation, with the experts operating in isolation from one another to prevent bias.

The finalized questions were submitted to ChatGPT-4 on the specified date, and the responses were collected for evaluation. The evaluation of these responses was conducted using the DISCERN scoring system, a reliable tool for assessing the quality of written health information. The DISCERN scoring system is comprised of sixteen questions, organized into three sections. The first section contains eight questions that assess the reliability of the information source, while the second section includes seven questions focused on the presentation of treatment options. The final single question evaluates the overall quality of the information provided. This scoring structure is specifically tailored to analyze the quality and usability of information related to treatment choices. In this study, the scores are categorized into five levels: very poor (16–26 points), poor (27–38 points), fair (39–50 points), good (51–62 points), and excellent (63–80 points) (7). This range allows for a nuanced evaluation of the information, ensuring it meets rigorous standards necessary for effective patient education and informed decision-making. The independent assessments by the three experts were then averaged to obtain the final DISCERN scores for each response.

In this study, various readability indices were employed to assess the comprehensibility of the responses provided by ChatGPT-4. The Flesch Reading Ease Score (FRES), developed in 1948, is one of the oldest and most widely used readability tools. It is commonly utilized to evaluate reading materials written for adults and is frequently applied in assessing medical literature due to its effectiveness in conveying complex information in an accessible manner. The Flesch-Kincaid Grade Level (FKGL) is typically used for evaluating secondary education materials and easily converts to U.S. grade levels, making it a standard tool in many organizations for document standardization. The Gunning Fog Index (GFOG) calculates readability by using the count of polysyllabic words and the average sentence length in a 100-word selected passage. Similarly, the Simple Measure of Gobbledygook (SMOG) uses the same variables but assesses the entire text, which is recommended by the National Cancer Institute for health information assessments. Developed in 1969, SMOG has been validated through studies showing a strong correlation with required document reading levels. The Coleman-Liau Index determines the grade level of a written document based on sentence length and character count. The Automated Readability Index (ARI) utilizes characters, word counts, and sentence counts to calculate readability (8–10). These indices were chosen because they facilitate comparisons with prior literature in otolaryngology, where these tools are predominantly utilized to evaluate readability levels (11–14). Additionally, readability is a critical component of health literacy, and various readability formulas are used to determine the reading ability required to comprehend a text. The American Medical Association (AMA) and the National Institutes of Health (NIH) recommend that patient education materials be written at or below the sixth-grade reading level to be accessible to the average adult in the United States, who reads at approximately the eighth-grade level (15–17). This recommendation is reflected in our analysis criteria, ensuring that the accessibility of the AI-generated responses is objectively measured and aligned with established health communication standards. These readability scores were calculated by transferring the texts to the website https://www.webfx.com/tools/read-able/, which automatically evaluates the comprehensibility of written materials. This automated approach provided an objective assessment of the texts' accessibility to the average reader.

Given that the study involved publicly available data and did not include human participants, ethical committee approval was not required. The study was conducted in accordance with relevant guidelines and regulations, ensuring the confidentiality and integrity of the data throughout the research process.

### Statistical analysis

Mean, standard deviation, median, minimum, maximum value frequency and percentage were used for descriptive statistics. The distribution of variables was checked with the Kolmogorov-Simirnov Test. Independent Samples *T*-Test was used for the comparison of quantitative data. Friedman Test was used for the repeated measurement analysis. Pearson Correlation Analysis was used in correlation analysis. SPSS 28.0 was used for statistical analyses.

## Results

In this study, fifteen hypothetical questions about otosclerosis surgery were posed to ChatGPT-4, and the responses were evaluated by an expert panel using the DISCERN scoring system to assess the quality of the health information provided. The scores awarded varied, with one response classified as poor (6.7%), six as fair (40%), six as good (40%), and two as excellent (13.3%). The average DISCERN score was 50.7 ± 8.2, indicating a moderate overall quality of the AI-generated advice regarding otosclerosis surgery (Table 1).

**Table 1:** DISCERN analysis summary for ChatGPT-4's otosclerosis consultations.

		Discern scores				Discern category
		Expert 1	Expert 2	Expert 3	Mean Scores	
Question 1	What is the success rate of otosclerosis surgery?	51	60	62	57,67	Good
Question 2	What are the risks of otosclerosis surgery?	41	44	50	45,00	Middle
Question 3	What type of anesthesia will be applied during otosclerosis surgery?	38	43	52	44,33	Middle
Question 4	How many days will I stay in the hospital after otosclerosis surgery?	48	51	57	52,00	Good
Question 5	When can I take a bath after otosclerosis surgery?	50	55	61	55,33	Good
Question 6	Will I have pain after otosclerosis surgery?	61	63	67	63,67	Perfect
Question 7	Will I need to use antibiotics after otosclerosis surgery?	48	41	52	47,00	Middle
Question 8	When can I return to work after otosclerosis surgery?	41	45	36	40,67	Middle
Question 9	Will I have a dressing in my ear after otosclerosis surgery?	42	32	36	36,67	Weak
Question 10	When will I start hearing normally after otosclerosis surgery?	49	43	51	47,67	Middle
Question 11	Will I need to use a device after otosclerosis surgery?	53	50	58	53,67	Good
Question 12	I had otosclerosis surgery, and blood started coming from my ear. What should I do?	56	63	59	59,33	Good
Question 13	I had otosclerosis surgery, is it normal for me to feel dizzy?	47	39	44	43,33	Middle
Question 14	I had otosclerosis surgery, when can I start swimming?	49	55	52	52,00	Good
Question 15	Which treatment is better for otosclerosis patients? Surgery or hearing aids?	63	65	63	63,67	Perfect
	Total DISCERN score (16–80)		*n*(15)		Mean ± sd/%	
	Very poor (16–26)		0		0%	
	Poor (27–38)		1		6,70%	
	Fair (39–50)		6		40%	
	Good (51–62)		6		40%	
	Excellent (63–80)		2		13,30%	
	Average score				50.7 ± 8.2	

The consistency among the three independent ENT specialists was high, as evidenced by an intraclass correlation coefficient (ICC) of 0.949, suggesting excellent agreement (*p* = 0.000). This high level of agreement underscores that, despite the subjective nature of the scoring system, there was significant concordance among the evaluators in assessing the quality of responses (Table 2).

**Table 2:** Intraclass correlation.

		1. Measurement	2. Measurement	3. Measurement	*p*¹	ICC	
						*r*	*p* ^ICC^
Discern	Mean ± sd	50.1 ± 7.3	50.3 ± 9.2	51.9 ± 9.1	0.119^F^	0.949	***0***.***000***^ICC^
	Median	49.0	50.0	52.0			

*p*^¹ F^ Friedman *t* test.

*p*^ICC^ (Intraclass Correlation).

The readability of the responses was assessed using various indices to evaluate how accessible the information would be to a general audience. The Flesch Reading Ease (FRE) indicated scores ranging from 20.6–48.5, with a mean of 34.9 ± 7.3, suggesting that the material is generally difficult to read and best suited for college-level readers. The Flesch-Kincaid Grade Level (FKGL) showed that the information requires the reading ability of at least a U.S. high school graduate or college student, with a mean grade level of 13.5 ± 1.6. The Gunning Fog Index, with a mean score of 16.4 ± 1.6, indicates that the content is appropriate for someone with some college education, due to the use of complex words and sentence structures. The Simple Measure of Gobbledygook (SMOG) and the Coleman-Liau Index suggest that the content is best understood by high school seniors or early college students, with average scores of 12.3 ± 1.2 and 15.3 ± 1.0, respectively. Lastly, the Automated Readability Index (ARI) provides a mean score of 13.6 ± 1.9, aligning with the readability for upper high school levels (Table 3). These indices collectively demonstrate that while the responses cover necessary and relevant topics regarding otosclerosis surgery, they are written at a complexity level potentially excluding those with lower educational attainment and do not meet the recommended standards for patient education materials, which should ideally be accessible at the sixth-grade reading level.

**Table 3:** Readability scores.

	Min-Max	Median	Mean ± sd.
FRE	20.6–48.5	35.5	34.9 ± 7.3
FKGL	10.3–15.7	13.8	13.5 ± 1.6
Gunning FOG	13.3–18.6	16.5	16.4 ± 1.6
SMOG	9.7–14.0	12.3	12.3 ± 1.2
CLI	13.8–17.6	15.5	15.3 ± 1.0
ARI	9.9–16.1	14.3	13.6 ± 1.9

## Discussion

In our study, over 90% of the responses provided by ChatGPT-4 were rated above the “poor” level, demonstrating the AI's effectiveness in delivering accurate and pertinent advice on otosclerosis surgery. These responses were categorized as “fair” (40%), “good” (40%), and “excellent” (13.3%), clearly indicating that the majority were considered satisfactory according to our expert panel's DISCERN scores. The only response that was rated “poor” (6.7%) was to the question “Will I have a dressing in my ear after otosclerosis surgery?” Although this response contained generally accurate information about post-surgical care, it lacked specific applicability to otosclerosis, pinpointing a need for improvement in the AI's ability to provide context-specific medical advice. This example highlights the necessity of carefully evaluating AI-generated responses, particularly in medical settings where precise and contextually appropriate information is crucial. Additionally, the readability analysis revealed that the text complexity might not be suitable for all readers, suggesting that adjustments are needed to make AI-generated health information more universally understandable.

This indicates the potential of ChatGPT to provide generally reliable and high-quality information. However, the low scores of some responses also indicate that the AI system can sometimes produce incomplete or misleading information. These results support the idea that AI-based systems should always be used under the supervision of human experts.

The readability analysis in our study showed that the texts produced by ChatGPT are not comprehensible to the average reader. This could be a barrier, especially for patients with lower education levels or low health literacy, as the comprehensibility of all the texts is above the 6th grade level. Therefore, additional adjustments are needed to make the content produced by AI systems simpler and more accessible.

While studies on the use of ChatGPT in healthcare show varied outcomes, there is a consensus that the readability often exceeds the optimal level for patient comprehension. For instance, Ulusoy et al. observed that ChatGPT's DISCERN scores were similar across different medical queries such as low back pain and meniscal injury, with relatively better readability than in otology (18). Seth et al. evaluated responses from three different AIs on rhinoplasty and noted that while the readability was high, the quality of information did not meet the desired standards (19). Further research in fields like periodontal disease and urology has demonstrated that while ChatGPT can provide useful guidance, it often lacks necessary sourcing, which is crucial for clinical reliability (20, 21). Additionally, a study involving cancer-related inquiries showed that AI could offer valuable insights, but the accuracy and quality of the information must be rigorously improved to avoid misinformation, especially in critical care contexts (21). A study analyzed responses from two different AIs, including ChatGPT, to 117 questions from the American Cancer Society about cancer. The results showed generally good quality answers, highlighting the potential of AI chatbots to significantly contribute to providing cancer-related information. Nonetheless, the study highlighted the need for ongoing improvements in the quality of these AI-provided responses to ensure accuracy, particularly for critical topics like cancer prognostics, where misinformation could lead to confusion and emotional distress (22).

Results from various disciplines show that the capacity of ChatGPT to provide health information varies across different topics. A high level of readability may in some cases indicate that technical terminology or complex medical information is not sufficiently simplified, meaning that the information provided by ChatGPT is not accessible to all users. This suggests that ChatGPT needs to strike a careful balance in its presentation of health information. It must be both sufficiently understandable and meet the requirements of medical accuracy and detail. The studies reviewed showed that ChatGPT provides high quality information on some health topics, but in some cases this information is not sufficiently detailed or accurate. This is particularly evident in areas that require technical knowledge and expertise.

It seems that ChatGPT and similar AI-based chatbots have significant potential in health information delivery. However, continuous improvement is needed in terms of information quality, accuracy and user accessibility. In order for these technologies to be used effectively in the field of health, it is of great importance to present information both in terms of accuracy and in a way that users can easily understand. While the reviewed studies are an important guide in the development of AI-based health information delivery, they also clearly reveal the improvements that need to be made in this field and the limitations that need to be taken into account. The results of these studies, while evaluating the overall performance of ChatGPT in health information delivery, draw attention to the areas that need to be improved and determine the steps needed to use these technologies more effectively and reliably in the field of health.

Recent studies have significantly contributed to understanding the potential and limitations of AI in otolaryngology, emphasizing the need for rigorous validation of AI tools before their integration into clinical practice. For instance, the development and validation of the QAMAI tool demonstrate a systematic approach to evaluate AI-generated health information, showing robust construct validity and high internal consistency which could be instrumental in ensuring the reliability of AI platforms, including ChatGPT, within otolaryngology settings (23). Furthermore, the complexity of using AI for synthesizing clinical guidelines is highlighted by the variability in AI responses compared to expert consensus, underscoring the necessity for AI to be used with caution, particularly in complex medical fields like otolaryngology (24). Moreover, studies exploring the potential of ChatGPT in specific otolaryngological applications such as obstructive sleep apnea management and sialendoscopy clinical decision-making reveal that while ChatGPT can align closely with expert opinions, discrepancies in clinical judgment call for a blended approach where AI supports rather than replaces human expertise (25, 26). Finally, the debate on whether AI can replace specialist doctors is ongoing. Despite AI's high correctness in responses, the variability and occasional inaccuracy, particularly in complex cases, highlight the irreplaceable value of human expertise in clinical decision-making (27).

These studies collectively illustrate the burgeoning role of AI in otolaryngology, advocating for a cautious yet optimistic approach towards its integration into clinical practice. By continuously refining AI capabilities and ensuring rigorous validation, AI can significantly augment otolaryngological care, enhancing diagnostics and patient education while maintaining the essential human touch in healthcare.

While this study has highlighted the potential of ChatGPT in providing accurate health information, one significant limitation is the model's inability to provide direct references for the information it generates. This is a crucial concern in the medical field where validation of information against credible sources is essential for clinical decision-making and patient education. Unlike traditional health information sources that cite references for verification, ChatGPT generates responses based on a trained dataset without the capability to link back to specific evidential sources. This limitation raises questions about the reliability and traceability of the information provided, which is vital for ensuring the safety and well-being of patients who might rely on such AI-generated advice.

The absence of direct citations makes it challenging for users to verify the currentness and accuracy of the information, potentially leading to decisions based on outdated or incorrect data. Future implementations of AI in healthcare could benefit from integrating systems that not only provide accurate responses but also include references or indications of the sources used in their training processes. This integration could enhance the credibility of AI applications in healthcare and increase their utility by enabling users to cross-check and validate the information with trusted medical literature.

When we look at the results in the literature, there seems to be no consensus on the quality of the responses given by the ChatGPT. In addition, the fact that ChatGPT does not specify the source, provides incomplete and incorrect information on some issues, and the readability level is far from the desired level shows that ChatGPT is not yet at the desired level in terms of patient information and needs to be improved. Consultation by an ENT specialist may still be the golden standard.

In conclusion, the potential of ChatGPT in providing health information should be carefully examined and continuously improved in terms of information quality and comprehensibility. The increasing use of this technology in healthcare is expected to have positive impacts on patient care and education. However, it is vital that these developments are evaluated and managed from the perspective of healthcare professionals and patient safety.

A limited number of questions may be a limitation to evaluate the performance of ChatGPT more comprehensively. Second, the datasets used in the training of ChatGPT include information up to a certain date, which may result in the most recent information or studies are not being addressed in the study. Finally, this research focuses specifically on the capacity of ChatGPT-4 to provide health information and the results may be specific to this model only. Similar research on other AI systems will likely yield different results as they have different features and capacities.

Further research is needed on how systems like ChatGPT can be optimized for patient education and health literacy improvement. Furthermore, while the increased use of these technologies in the healthcare sector is expected to have positive impacts on patient care and education, it is vital that these developments are carefully evaluated and managed for healthcare professionals and patient safety.

## Conclusion

Our evaluation of ChatGPT-4's effectiveness as a digital health advisor for otosclerosis surgery shows that while it accurately delivers health information, as indicated by mostly fair to excellent DISCERN scores, its utility is limited by the high readability level of its responses, which are not easily comprehensible to the average reader. Furthermore, the potential for incomplete or misleading information highlights the need for continuous improvement and close supervision by healthcare professionals to ensure the safe and effective use of AI in patient education. As AI technologies evolve, they promise to enhance healthcare delivery by providing accessible information; however, to realize this potential fully, ongoing development, guided by collaborative efforts between AI developers, healthcare providers, and policymakers, is essential to establish standards that ensure the information's reliability and ethical integrity.

## Data Availability

The raw data supporting the conclusions of this article will be made available by the authors, without undue reservation.
